# Recombination Marks the Evolutionary Dynamics of a Recently Endogenized Retrovirus

**DOI:** 10.1093/molbev/msab252

**Published:** 2021-09-04

**Authors:** Lei Yang, Raunaq Malhotra, Rayan Chikhi, Daniel Elleder, Theodora Kaiser, Jesse Rong, Paul Medvedev, Mary Poss

**Affiliations:** 1 Department of Biology, The Pennsylvania State University, University Park, PA, USA; 2 Center for Comparative Genomics and Bioinformatics, The Pennsylvania State University, University Park, PA, USA; 3 Department of Computer Science and Engineering, The Pennsylvania State University, University Park, PA, USA; 4 Department of Biochemistry and Molecular Biology, The Pennsylvania State University, University Park, PA, USA; 5 Institute of Molecular Genetics, Academy of Sciences of the Czech Republic, Prague, Czech Republic

**Keywords:** endogenous retrovirus, CrERV, recombination, genome diversity, mule deer, insertional polymorphism

## Abstract

All vertebrate genomes have been colonized by retroviruses along their evolutionary trajectory. Although endogenous retroviruses (ERVs) can contribute important physiological functions to contemporary hosts, such benefits are attributed to long-term coevolution of ERV and host because germline infections are rare and expansion is slow, and because the host effectively silences them. The genomes of several outbred species including mule deer (*Odocoileus hemionus*) are currently being colonized by ERVs, which provides an opportunity to study ERV dynamics at a time when few are fixed. We previously established the locus-specific distribution of cervid ERV (CrERV) in populations of mule deer. In this study, we determine the molecular evolutionary processes acting on CrERV at each locus in the context of phylogenetic origin, genome location, and population prevalence. A mule deer genome was de novo assembled from short- and long-insert mate pair reads and CrERV sequence generated at each locus. We report that CrERV composition and diversity have recently measurably increased by horizontal acquisition of a new retrovirus lineage. This new lineage has further expanded CrERV burden and CrERV genomic diversity by activating and recombining with existing CrERV. Resulting interlineage recombinants then endogenize and subsequently expand. CrERV loci are significantly closer to genes than expected if integration were random and gene proximity might explain the recent expansion of one recombinant CrERV lineage. Thus, in mule deer, retroviral colonization is a dynamic period in the molecular evolution of CrERV that also provides a burst of genomic diversity to the host population.

## Introduction

Retroviruses are unique among viruses in adopting life history strategies that enable them to exist independently as an infectious RNA virus (exogenous retrovirus, XRV) (Coffin 1996) or as an integral component of their host germline (endogenous retrovirus, ERV) (Löwer et al. 1996; Weiss 2006). An ERV is the result of a rare infection of a germ cell by an XRV and is maintained in the population by vertical transmission. Germline colonization has been a successful strategy for retroviruses as they comprise up to 10% of most contemporary vertebrate genomes (Stoye 2012). Over the evolutionary history of the species, ERV composition increases by acquisition of new germ line XRV infections, and through retrotransposition or reinfection of existing ERVs (Boeke and Stoye 1997; Belshaw et al. 2004; Belshaw, Katzourakis, et al. 2005; Johnson 2015), which results in clusters of related ERVs. The ERV profile in extant species therefore reflects both the history of retrovirus epizootics and the fate of individual ERVs. Because the acquisition of retroviral DNA in a host genome has the potential to affect host phenotype (Jern and Coffin 2008; Kurth and Bannert 2010; Feschotte and Gilbert 2012), the dynamic interactions among ERVs and host could shape both retrovirus and host biology. However, the evolutionary processes in play near the time of colonization are difficult to discern based on an ERV colonization event that occurred in an ancestral species. A better understanding of both host and virus responses to recent germ line invasion might inform homeostatic changes in ERV-host regulation that are relevant to the pathogenesis of diseases in which ERV involvement has been implicated (Antony et al. 2011; Magiorkinis et al. 2013; Wildschutte et al. 2014; Li et al. 2015; Li, Yang, et al. 2019; Xue et al. 2020). Fortunately, there is now evidence that retrovirus colonization is occurring in contemporary, albeit often nonmodel, species (Arnaud et al. 2007; Elleder et al. 2012; Roca et al. 2017), allowing for investigation of ERV dynamics near the time of colonization. Our goal in this research is to investigate the evolutionary dynamics of phylogenetically distinct ERV lineages that have sequentially colonized mule deer over the approximate 1 My history of this species using the complete genome sequence of a majority of coding ERVs in the context of a draft assembly of a newly sequenced mule deer genome.

The life history strategy adopted by retroviruses indicates why this virus family has been so successful in colonizing host germline. Retroviral replication requires that the viral RNA genome be converted to DNA and then integrated into the genome of an infected cell (Vogt 1997). As with many RNA viruses, the virus polymerase enzyme, reverse transcriptase (RT), is error prone, which contributes to a high mutation rate and enables rapid host adaptation. In addition, RT moves between the two RNA copies that comprise a retroviral genome (Luo and Taylor 1990); this process can repair small genomic defects and increases evolutionary rates via recombination if the two strands are not identical. Retroviral DNA is transported to the nucleus where it integrates into host genomic DNA using a viral integrase enzyme. The integrated retrovirus is called a provirus and represents a newly acquired gene that persists for the life of the cell and is passed to daughter cells, which for XRV are often hematopoietic cells. A retrovirus that infects and integrates into a germ cell is called an ERV. In this case all nucleated cells in an organism will contain the new retroviral DNA if reproduction of the infected host is successful.

The retroviral life cycle also demonstrates how ERVs can affect host biology (Jern and Coffin 2008; Bolinger and Boris-Lawrie 2009). ERVs require host transcription factors and RNA polymerases to bind to the retrovirus promoter, called long-terminal repeats (LTRs), to produce viral transcripts and the RNA genome. Thus, the viral LTRs compete with host genes for transcription factors and polymerases (Sofuku and Honda 2018). A retrovirus encodes at a minimum, genes for the capsid, viral enzymes, and an envelope gene needed for cell entry, which is produced by a subgenomic mRNA. Hence an ERV also utilizes host-splicing machinery and can alter host gene expression pattern if the site of integration is intronic (Isbel and Whitelaw 2012; Kim 2012). Although XRVs are expressed from small numbers of somatic cells, ERVs are present in all nucleated cells and ERV transcripts and proteins can be expressed in any cell type at any stage of host development. Hosts actively silence the expression of full or partial ERV sequences by epigenetic methods (Yao et al. 2004; Hurst and Magiorkinis 2017) and by genes encoding viral restriction factors (Lavie et al. 2005; Matsui et al. 2010; Sze et al. 2013; Bruno et al. 2019; Geis and Goff 2020). Because there will be no record of an ERV that causes reproductive failure of the newly colonized host, ERVs in contemporary vertebrates are either effectively controlled by host actions, are nearly neutral in effects on host fitness, or potentially contribute to the overall fitness of the host (Haig 2012; Göke and Ng 2016; Blanco-Melo et al. 2017; Fu et al. 2019).

The coding portion of a new ERV can be eliminated from the genome through nonallelic homologous recombination (NAHR) between the LTRs, which are identical regions that flank the viral coding portion. A single LTR is left at the site of integration as a consequence of the recombination event and serves as a marker of the original retrovirus integration site (Hughes and Coffin 2004). Most ERV integration sites in humans are solo LTRs (Belshaw, Dawson, et al. 2005; Subramanian et al. 2011). Because the efficiency of NAHR is highest between identical sequences (Hoang et al. 2010), conversion of a full-length ERV to a solo LTR likely arises early during ERV residency in the genome before sequence identity of the LTR is lost as mutations accrue (Belshaw et al. 2007). Because mutations are reported to arise in ERVs at the neutral mutation rate of the host (Kijima and Innan 2010), sequence differences between the 5′ and 3′ LTR of an ERV have been used to approximate the date of integration (Johnson and Coffin 1999; Zhuo et al. 2013).

Although in humans most ERV colonization events occurred in ancestral species, acquisition of new retroviral elements is an ongoing (Stocking and Kozak 2008; Anai et al. 2012) or contemporary (Roca et al. 2017) event in several animal species. The consequences of a recent ERV acquisition are important to the host species because it creates an insertionally polymorphic site; the site is occupied in some individuals but not in others. All ERVs are insertionally polymorphic during the trajectory from initial acquisition to fixation or loss in the genome. Indeed, the human ERV type K (HERV-K) family is insertionally polymorphic in humans (Soriano et al. 1987; Turner et al. 2001; Moyes et al. 2007; Wildschutte et al. 2016) and HERV-K prevalence at polymorphic sites differs among global populations (Li, Lin, et al. 2019). Phylogenetic analyses of the ERV population in a genome can reveal the origins of ERV lineages, determine which are actively expanding in the genome, when and how often expansion of an ERV occurs, and the mutational processes that drive evolution. These data indicate if expansion is related to the site of integration or a feature of the virus, or both and coupled with information of ERV prevalence at insertionally polymorphic sites, can inform ERV effects on host phenotype.

To this end, we explored the evolutionary history of the mule deer (*Odocoileus hemionus*) ERV (Cervid ERV, CrERV; a gammaretrovirus) because we have extensive data for prevalence of CrERV loci in northwestern US mule deer populations (Bao et al. 2014; Kamath et al. 2014) and preliminary data on CrERV sequence variation and colonization history (Elleder et al. 2012; Kamath et al. 2014). A majority of CrERV loci is insertionally polymorphic in mule deer; 90% of animals shared fewer than ten of approximately 250 CrERV integrations per genome in one study (Bao et al. 2014). Further, based on the sequence of CrERV identified in several mule deer, at least four distinct lineages have been successful in germ line colonization (Kamath et al. 2014). Because none of the CrERV loci occupied in mule deer are found in the sister species, white-tailed deer (*Odocoileus virginianus*) (Elleder et al. 2012), all endogenization events have likely occurred since the split of these sister taxa. A full-length retrovirus representing the youngest of the CrERV lineages was recovered by coculture on human cells, indicating that some CrERV are still capable of infection (Fábryová et al. 2015). In this study, we expand on these preliminary data by sequencing a mule deer genome and conducting phylogenetic analyses on a majority of reconstructed CrERV genomes. Our results demonstrate that expression and recombination of recently acquired CrERV with older CrERV have increased CrERV burden and diversity and consequently have increased contemporary mule deer genome diversity.

## Results

### Establishing a Draft Mule Deer Reference Genome to Study CrERV Evolution and Integration Site Preference

We developed a draft assembly of a mule deer genome from animal MT273, in order to determine the sequence at each CrERV locus for phylogenetic analyses and to investigate the effect of CrERV lineage or age on integration site preference. ERV sequences are available in any genome sequencing data because a retrovirus integrates a DNA copy into the host genome. However, the most recently integrated ERVs are nearly identical making them difficult to assemble and causing scaffolds to break at the site of an ERV insertion (Chaisson et al. 2015). We assembled scaffolds using a combination of high-coverage Illumina short-read whole-genome sequencing (WGS) and long-insert mate pair sequencing. Our de novo assembly yielded an ∼3.31 Gb draft genome with an N50 of 156 kb (supplementary table S1, Supplementary Material online), which is comparable to the 3.33 Gb (*c* value of 3.41 pg) experimentally determined genome size of reindeer (*Rangifer tarandus*) (Vinogradov 1998; Gregory 2019).

**Fig. 1.**
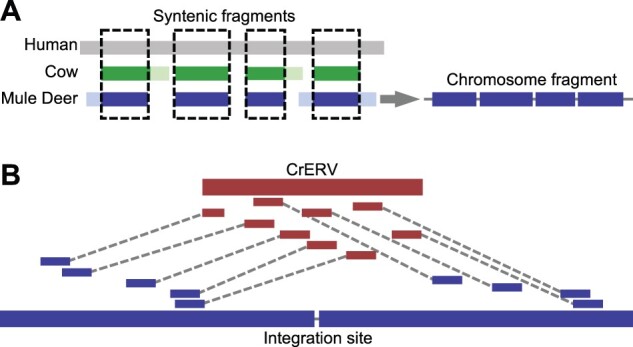
Diagram of CrERV reconstruction and RACA. (*A*) Mule deer chromosome fragment reconstruction using syntenic fragments. Gray, green, and blue boxes correspond to aligned human, cow, and mule deer scaffold respectively. Lighter shades represent regions that can only be aligned between two species. Dashed boxes highlight syntenic fragments where the region is conserved among all three species, which yield a chromosome fragment that orients mule deer scaffolds. (*B*) Reconstruction of CrERV sequences. CrERV and mule deer scaffolds are shown in bold red and blue boxes, respectively. Long-insert mate pair reads are connected by dotted lines and are colored to indicate whether they derive from the mule deer scaffold or CrERV genome. CrERV genomes were assembled by gathering the broken mate pairs surrounding each CrERV loci as described.

Approximately half of CrERV loci are located at the ends of scaffolds based on mapping our previously published junction fragment sequences (Bao et al. 2014), which is consistent with the fact that repetitive elements such as ERVs break scaffolds (Chaisson et al. 2015). To determine the sequence of these CrERVs and the genome context in which they are found, we developed a higher order assembly using reference-assisted chromosome assembly (RACA) (Kim, Larkin, et al. 2013). RACA further scaffolds our de novo mule deer assembly into “chromosome fragments” by identifying synteny blocks among the mule deer scaffolds, the reference species genome (cow), and the outgroup genome (human) (fig. 1*A*). We created a series of RACA assemblies based on scaffold length to make efficient use of all data (supplementary table S1, Supplementary Material online). RACA150K takes all scaffolds greater than 150,000 bp as input and yielded 41 chromosome fragments, 35 of which are greater than 1.5 Mb; this is consistent with the known mule deer karyotype of 2*n* = 70 (Gallagher et al. 1994). However, RACA150K only incorporates 48% of the total assembled sequences (1.59 Gb) because of the scaffold size constraint. In contrast, RACA10K uses all scaffolds 10,000 bp or longer and increases the assembly size to 2.37 Gb (∼72% of total assembly) but contains 658 chromosome fragments (supplementary table S1, Supplementary Material online). The majority of scaffolds that cannot be incorporated into a RACA assembly are close to the ends of alignment chains (supplementary file S1, section 1a, Supplementary Material online). Most sequences not represented in any assemblies were repeats based on *k-mer* analyses (supplementary file S1, section 1a and fig. S1, Supplementary Material online).

Some scaffolds were excluded from the RACA assemblies, presumably because there is no synteny between cow and human for these sequences. We oriented these scaffolds using the cow–mule deer and sheep–mule deer alignments (RACA+, supplementary table S2, Supplementary Material online). Approximately 124 Mb of sequence (∼4% of total assembly) is in scaffolds larger than 10 kb but cannot be placed in RACA10K, nearly all of which can be found on the mule deer–cow alignment chain and the mule deer–sheep (oviAri3) alignment chain (123 Mb in each chain). Because there is overlap between these alignments, only ∼1 Mb is specific to cow and ∼1 Mb is specific to sheep. Therefore, RACA+ incorporated all but 69 scaffolds that are greater than 10 kb, which consisted of 1.17 Mb of sequence (∼0.04% of total scaffold size of the assembly) and yields an assembly size of 2.49 Gb (supplementary table S1, Supplementary Material online).

To enable the investigation of CrERV integration site preference relative to host genes, we annotated the mule deer scaffolds. We used Maker2 (Cantarel et al. 2008; Holt and Yandell 2011) for the annotation, which detects candidate genes based on RNA sequencing data and protein homology to any of the three reference genomes: human, cow, and sheep. After four Maker iterations, 21,598 genes with an annotation edit distance (Cantarel et al. 2008) of less than 0.8 were annotated (supplementary table S3, Supplementary Material online). Approximately 92% of genes are found on RACA150K scaffolds and 95% of genes are represented in RACA10K scaffolds.

### Establishing the Location and Sequence at CrERV Loci

Several lines of evidence suggest that most CrERVs are missing from the assemblies. Only three CrERVs with coding potential were assembled by the de novo assembly. The *k-mer* based analysis shows that less than 9.62% of all LTR repeat elements are in the assemblies (supplementary table S4, Supplementary Material online). The CrERV-host junction fragments previously sequenced (Bao et al. 2014) support that CrERV loci are near scaffold ends or long stretches of “N’s.” Therefore, we took advantage of the different chromosome fragments generated by RACA10K, RACA150K, and RACA+ and the long-insert mate pair sequencing data to reconstruct CrERVs at each locus (fig. 1*B*). We identified 252 CrERV loci in the MT273 genome, which is consistent with our estimates of an average of 240 CrERV loci per mule deer by quantitative PCR (Elleder et al. 2012) and 262 CrERV loci in animal MT273 by junction fragment analysis (Bao et al. 2014). The majority of CrERV loci (206/252) contain CrERVs with some coding capacity and 46 are solo LTRs. Of the 206 CrERVs containing genes, 164 (supplementary table S6, Supplementary Material online) were sufficiently complete to allow phylogenetic analysis on the entire genome or, if a deletion was present, on a subset of viral genes; at 42 loci, we were unable to obtain sufficient lengths of high-quality data for further analyses. Consistent with the findings of Kamath et al. (2014), there are no differences between the 5′ and 3′ LTR sequence, which is often used to age ERV genome residency, in a majority of CrERV. Thus our more comprehensive phylogenetic approach based on genome sequence is needed to establish CrERV evolutionary history.

### Evolutionary History of CrERV

**Fig. 2.**
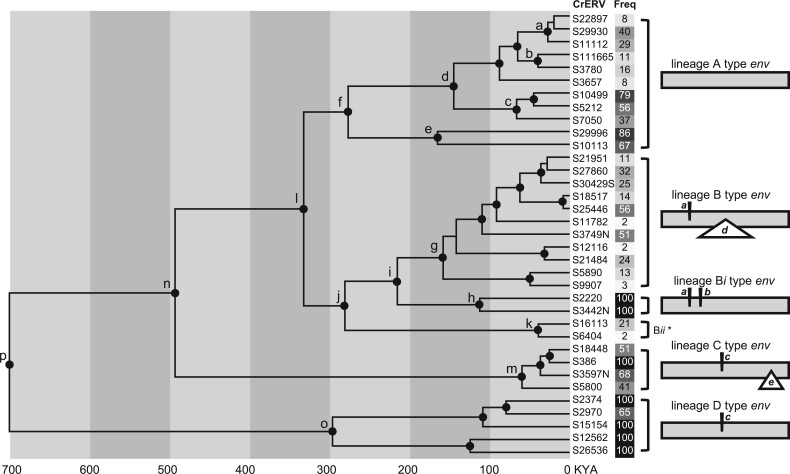
Coalescent phylogeny, *env* structural variation, and population frequency of representative full-length nonrecombinant CrERVs. This is an unrooted phylogeny based on the alignment of 34 CrERV sequences (1,477–8,633 relative to JN592050) with no signature of recombination. The region of the genome spanning a portion of *env* (6,923–7,503 bp relative to JN592050) was manually blocked to accommodate variable regions in different CrERV lineages. Nodes with at least 95% posterior probability support are marked by black dots. The high posterior density for each labeled node is shown in supplementary table S7, Supplementary Material online. The percentage of mule deer that carry a CrERV is given in a linear gray scale background (white = 0, black = 100) (see supplementary table S6, Supplementary Material online, for additional information). Diagrams on the right side depict the lineage-specific structural variations in the CrERV *env*. Insertions are represented by italicized lower case characters (*a*, *b, and c*) above the sequence; deletions are shown within a triangle under the sequence (*d and e*). *, a schematic for Lineage B*ii* could not be made because the five representatives of this lineage had incomplete coverage of *env*.

We previously showed that mule deer genomes have been colonized multiple times with the gammaretrovirus CrERV since the ancestral split with white-tailed deer approximately 1 Ma (Kamath et al. 2014) because none of the CrERV integration sites are found in white-tailed deer (Elleder et al. 2012). To better resolve the colonization history, we interrogated various combinations of CrERV alignments spanning position 1,477–8,633 relative to GenBank accession number JN592050 with PhiPack (Bruen et al. 2006) and identified 34 reconstructed CrERV sequences with high-quality data, no signature of recombination, and that were representative of the phylogenetic structure of a larger data set (supplementary table S5*A* and file S1, section 2i, Supplementary Material online). A coalescent analysis was conducted based on the 1,477–8,633 alignment of the 34 representative CrERVs (fig. 2). *Env* has regions that are highly variable and are not alignable because of large insertions or deletions interspersed between conserved regions, which typically represent regions of structural conservation but divergent sequence (Benit et al. 2001). To retain this information, the region of *env* spanning 6,923–7,503 bp (based on JN592050 coordinates) was manually blocked to accommodate the variable regions in the retrovirus *env*. The right panel of figure 2 depicts the pattern of *env* insertions and deletions characteristic of each lineage (see supplementary table S6, Supplementary Material online, column C for the *env* variable structure of each reconstructed CrERV). The resultant tree shows four well-supported CrERV lineages, each diverged from a common ancestor since the split of mule deer and white-tailed deer; these results are consistent with the phylogenetic structure of CrERV based on a partial genome alignment reported previously (Kamath et al. 2014).

Lineage A CrERVs encode a complete open reading frame for all retroviral genes. Our estimates indicate that the divergence of Lineage A and Lineage B ancestors occurred approximately 300 ka (fig. 2, node l). Lineage A represents 30% of all CrERV sampled from MT273 (table 1 and supplementary table S6, Supplementary Material online). There are several well-supported Lineage A CrERV subgroups that colonized mule deer genomes over the last 150 ky (fig. 2, nodes a–e; and supplementary table S7, Supplementary Material online). Our age estimates for each subgroup of Lineage A CrERV are consistent with their prevalence in populations of mule deer in the Northern Rocky Mountain ecosystem (fig. 2 and table 1); (Bao et al. 2014; Hunter et al. 2017). For example, S29996 and S10113 are the oldest Lineage A CrERV subgroup (fig. 2, node e) and occur in more than two-thirds of our sampled mule deer compared with those estimated to have entered the genome more recently (see S22897 and S111665, fig. 2, nodes a and b; TMRCA estimates for these subgroups are consistent with those of Kamath et al. [2014]). An infectious virus recovered by coculture belongs to the sublineage at node a (Fábryová et al. 2015).

**Table 1.** Summary of CrERV Lineages.

Lineage	*Env* Status	Prevalence (%)	Number of Loci
A	Full length	17.46	50
B	Insertion (*a*) and deletion (*d*)	14.29	52
B*i*	Insertion (*a and b*)	31.75	15
B*ii*	Missing data	11.11	5
C	Insertion (*c*) and deletion (*e*)	50.79	22
D	Insertion (*c*)	74.6	20

Notes.—*Env* status reflects the *env* structure of CrERV lineages as shown in figure 2. Prevalence shows the median frequency (percentage) of each CrERV lineage in 63 mule deer. Number of loci is based on CrERVs with sufficient data for their sequence reconstruction and used in our analyses.

Lineage B CrERV represents 32% of those sampled from our sequenced genome (table 1 and supplementary table S6, Supplementary Material online). Like Lineage A, the prevalence of most CrERV from Lineage B among mule deer in the northern Rockies region is low, reflecting their more recent colonization of the mule deer genome. Indeed, six Lineage B CrERVs were identified only in MT273, which could be indicative of a recent expansion (supplementary table S6, Supplementary Material online, column F). Lineage B CrERVs have a short insertion in the 5′ portion of *env* followed by a deletion that removes most of the *env* surface unit (SU) relative to Lineage A *env* while retaining the transmembrane region (TM) (fig. 2, insertion ***a***, deletion *d*). The phylogenetic history of Lineage B CrERV recorded in the mule deer genome indicates that all members that share this *env* structure arose approximately 150 ka (fig. 2, node g), but that there are two related groups of CrERV affiliated with Lineage B (Lineage B*i* and B*ii*; fig. 2 nodes j and i, table 1 and supplementary table S6, Supplementary Material online) with different *env* structures. Lineage B*i* shares the short 5′ insertion “*a*” in *env* but has a full-length *env* with an additional short insertion (insertion *b*) relative to the *env* of Lineage A CrERV. CrERVs with this *env* configuration represent 9% of coding CrERV in MT273. Because the prevalence of Lineage B*i* is high in mule deer, this group could represent the ancestral state for Lineage B CrERVs. The second group appears to have a unique *env* not found in any other CrERV lineages (Lineage B*ii*, fig. 2, node k; S16113 and S6404). We were able to estimate the prevalence for only two of these five unusual *env*-containing CrERV in mule deer because the host junction fragments for the other three are not represented in our draft mule deer assembly. The *env* sequence was incomplete for all representatives so we were unable to reconstruct the complete *env* of any of the five CrERV in this group. It is possible that these retroviruses evolved in a different species and represent a cross-species infection; it would be interesting to determine if representatives of Lineage B*ii* are found in the genomes of other species that occupied the ecosystem in the past.

There are 22 (13%) CrERV in the data set that have the signature 59 bp insertion “*c*” and 362 bp deletion “*e*” in *env* (fig. 2; supplementary table S6 and fig. S2, Supplementary Material online) compared with the full-length *env* of Lineage A; none have an intact *env* ORF. Of the CrERV affiliated with this lineage four met our criteria of having high-quality full-length sequence and no signature of recombination. Our coalescent estimates date the common ancestor of Lineage C CrERV to about 500 ka (fig. 2 and supplementary table S7, Supplementary Material online). Consistent with a longer residence in the genome one Lineage C CrERV is found in all mule deer sampled and the other three in more than 40% of animals (fig. 2 and supplementary table S6, Supplementary Material online, column F). However, these four CrERV share a common ancestor ∼50 ka (95% HPD: 16–116 ka, supplementary table S7, Supplementary Material online), which is consistent with a recent expansion of a long-term resident CrERV.

The first representatives of the CrERV family still identifiable in mule deer colonized shortly after their split from white-tailed deer, approximately 1 Ma (Elleder et al. 2012; Kamath et al. 2014). Lineage D CrERVs comprise 12% of reconstructed CrERV in MT273 and appear to be near fixation. Indeed, all mule deer in a larger survey of over 250 deer had CrERV S26536, which is not found in white-tailed deer (Kamath et al. 2014). This lineage shares *env* insertion “c” with Lineage C but lacks deletion “e,” (fig. 2 and supplementary fig. S2, Supplementary Material online) which removes the TM of *env*.

### Recombination among CrERV Lineages

Our coalescent estimates (fig. 2 and supplementary table S7, Supplementary Material online) indicate that two phylogenetically distinct CrERV lineages have been expanding in contemporary mule deer genomes over the last 100,000 years. Although CrERVs represented by Lineage A are capable of infection (Fábryová et al. 2015), all Lineage B CrERVs have an identical deletion of the SU portion of *env* and should not be able to spread by reinfecting germ cells. However, the mule deer genome comprised approximately equal percentages of Lineage B and Lineage A CrERVs so we considered two modes by which defective Lineage B CrERVs could expand in the genome at a similar rate with Lineage A. Firstly, ERVs that have lost *env* are proposed to preferentially expand by retrotransposition (Gifford et al. 2012) because a functional envelope is not necessary for intracellular replication. Secondly, we consider that Lineage B CrERVs could increase in the genome by infection if the cocirculating Lineage A group provided a functional envelope protein, a process called complementation (Mager and Freeman 1995; Belshaw, Katzourakis, et al. 2005) This latter mechanism requires that a member of each CrERV lineage be transcriptionally active at the same time in the same cell, and that intact proteins from the “helper” genome be used to assemble a particle with a functional envelope for reinfection. If two different CrERV loci are expressed in the same cell, either or both genomes could be copackaged in the particle. Because the RT moves between the two RNA genomes as first strand DNA synthesis proceeds, evidence of interlineage recombination would support that the molecular components necessary for complementation were in place. We assessed Lineage B CrERV for recombination with Lineage A to determine if coincident expression of the RNA genomes of these two lineages could explain the expansion by infection through complementation of the *env*-less Lineage B CrERV.

**Fig. 3.**
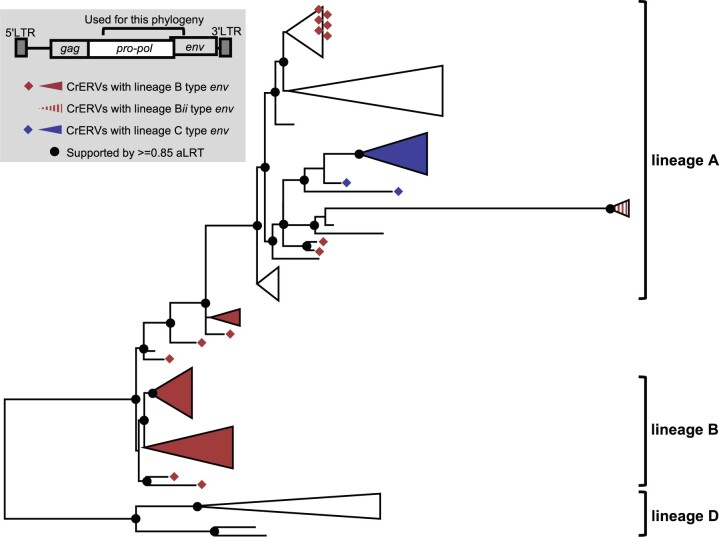
Recombination among CrERVs. Shown is a maximum likelihood phylogeny based on a region spanning a portion of *pol* to 5′*env* (JN592050: 4,422–7,076). Taxa used are a subset of full-length nonrecombinant CrERVs representing the four lineages shown in figure 2 and CrERVs with a recombinant signature containing a Lineage B *env*. Supported nodes (aLRT≥0.85) are represented by black dots on the backbone of the tree. Lineage designation is assigned to supported branches based on the nonrecombinant CrERV. Over this interval, Lineage B CrERVs are found as a sister group to Lineage A CrERV but some CrERV containing a prototypical Lineage B *env* are dispersed among Lineage A CrERV. Note that in this interval Lineage C CrERVs cluster with Lineage A CrERVs.

There is good support for recombination between Lineages A and B in a region spanning a portion of *pol* to the beginning of the variable region in *env* (4,422–7,076 based on coordinates of JN592050). In this region, several CrERV, which we provisionally classified as Lineage B because they carried the prototypical *env* deletion “*d*” of SU form a monophyletic group that is affiliated with Lineage A CrERV (fig. 3, upper collapsed clade containing red diamonds). By scanning the alignments between the recombinant and nonrecombinant CrERVs using PhiPack (Bruen et al. 2006) (supplementary table S5*B*, Supplementary Material online), we found that these Lineage B recombinants all share the same recombination breakpoint just 5′ of the characteristic short insertion “a” for these viruses (supplementary fig. S3, Supplementary Material online, indicated by “**” and supplementary table S5*B*, Supplementary Material online). In addition, several other CrERVs with Lineage B *env* branch between Lineages A and B, indicating that the recombination breakpoints fall within the region assessed (supplementary fig. S3, Supplementary Material online). Indeed, the breakpoint in a group of three A_B recombinant CrERV is at position 6630 based on coordinates of JN592050, which is near the predicted splice site for *env* at position 6591 (Elleder et al. 2012); this confers an additional 500 bp of the Lineage B *env* on these viruses (supplementary fig. S3, Supplementary Material online) resulting in their observed phylogenetic placement. Because recombination between the two retroviral RNA genomes occurs during reverse transcription, our data support that both Lineage A and B CrERVs were expressed and assembled in a particle containing a copy of each genome. A functional envelope protein from a Lineage A CrERV would therefore have been available for infection. These data are consistent with our premise that complementation by a replication competent Lineage A CrERV or CrXRV (cervid exogenous gammaretrovirus, an exogenous version of CrERV) contributes to the 32% prevalence of *env*-deleted Lineage B CrERV in the genome. It is likely that retrotransposition of the newly integrated Lineage A–B recombinant CrERV occurred because the clusters all share the same recombination breakpoint and the sequences are nearly identical (fig. 3, red diamonds in the Lineage A type *env* cluster).

There is additional data to support that Lineage B CrERV were transcriptional activity, which is requisite for recombination with an infectious Lineage A CrERV or for retrotransposition. We identified a nonrecombinant Lineage B CrERV (S24870 in supplementary table S6, Supplementary Material online) with extensive G to A changes (184 changes) compared with other members of this monophyletic group. These data are indicative of a cytidine deaminase acting on the single-stranded DNA produced during reverse transcription (Suspène et al. 2004).

Lineage C CrERV are enigmatic because, based on full-length sequences lacking a signature of recombination, its most recent common ancestor is estimated to emerge around 500 ka (fig. 2) but all extant members of this group share a common ancestor approximately 50 ka. From figure 3, it is evident that over the region of *pol* assessed, CrERVs containing the Lineage C *env* cluster with a Lineage A subgroup (represented by node e in fig. 2). The *env* of Lineage C CrERV shares sequence identity and insertion “*c*” with that of the Lineage D (supplementary fig. S2, Supplementary Material online), suggesting that Lineage C is in fact the result of recombination between an older representative of Lineage A (fig. 2, node e and supplementary table S6, Supplementary Material online) and a relative of a Lineage D CrERV. Although one Lineage C CrERV is fixed in the sampled mule deer, nine are found in 50–80% of animals, which is similar to the prevalence of the subgroup of Lineage A CrERV most closely related to Lineage C in *pol* (fig. 2 and supplementary table S6, Supplementary Material online, column F). These data are consistent with an initial interlineage recombination event occurring during the first wave of Lineage A CrERV colonization. Fourteen of the 22 CrERV in Lineage C have multiple signatures of recombination predominantly with Lineage A CrERV (supplementary table S6, Supplementary Material online, column D); some recombinants carry partial Lineage A *env* sequence that ablates insertion “c” or restores deletion “e.” The expansion of a subset of Lineage C as a monophyletic group approximately 50 ka (fig. 2 and supplementary table S7, Supplementary Material online) suggests that, like some members of Lineage B, CrERVs generated by recombination with Lineage A were transcriptionally active, recombinants integrated, and subsequently expanded in the genome. Our designation of Lineage C as derived from a nonrecombinant CrXRV is therefore incorrect. Instead, Lineage C CrERVs are derived from a CrERV or CrXRV that is not currently represented in mule deer genomes either because it was lost or it never endogenized, or because the identity of the parental CrERV has been obscured by multiple rounds of recombination. This CrERV subsequently was activated and recombined as Lineage A colonized the mule deer genome.

### Genomic Distribution of CrERV Lineages

Of the 164 CrERV that we reconstructed from MT273, only 12 can be detected in all mule deer that we have sampled (Bao et al. 2014; Kamath et al. 2014) (supplementary table S6, Supplementary Material online, column F). This means that the majority of CrERV loci in mule deer are insertionally polymorphic; not all animals will have a CrERV occupying a given locus. ERVs can impact genome function in multiple ways but the best documented is by altering host gene regulation, which occurs if the integration site is near a host gene (Rebollo et al. 2012). Thus, we investigated the spatial distribution of CrERV loci relative to host genes to determine the potential of either fixed or polymorphic CrERV to impact gene expression, which could affect host phenotype.

**Fig. 4.**
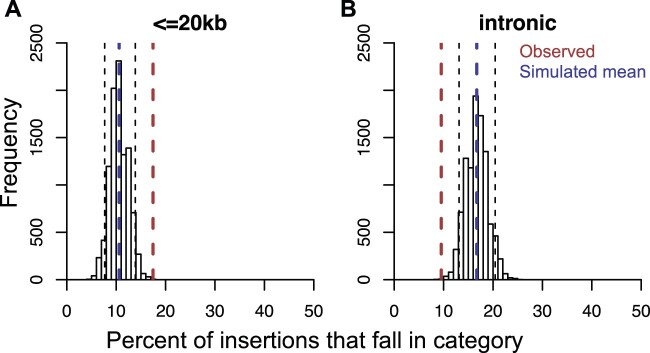
CrERV insertions are enriched within 20 kb of genes and depleted in introns. We determined the expected number of CrERV insertions near genes if integrations were random by simulation using the de novo assembled MT273 genome. The proportion of random insertions expected within 20 kb of a gene from the 10,000 replicates is shown in panel (*A*). The proportion of intronic insertions is in panel (*B*). The distribution of insertions within 20 kb of a gene or an intron from the simulation is shown as a histogram. Blue dashed lines indicate the mean of the simulated data. Red dashed lines indicate the observed data in MT273. Black dashed lines indicate the 5th and 95th percentile of the simulated data, which are used to call significant differences.

We considered that the actual distance of any point to a gene is likely to be unreliable in our assembly because most high copy number repeats are missing in the mule deer assembly (supplementary fig. S1, table S4, and section 1a of file S1, Supplementary Material online). Although there is no expectation that retrovirus insertion is random (Desfarges and Ciuffi 2010), we simulated a random distribution of retrovirus insertions (supplementary file S1, section 2l, Supplementary Material online) as a means to compare distributions in mule deer (scaffold N50 = 156 kb) with the genomes of cow (Btau7, scaffold N50 = 2.60 Mb) and human (hg19, scaffold N50 = 46.4 Mb). The mean distance between an insertion and the closest gene for all simulation replicates (supplementary fig. S4, Supplementary Material online) is significantly higher in the cow and human (Mann–Whitney *U* test *P *<* *2.2×10^−16^ for any pair of comparison among the three species). Therefore, to determine if any CrERV had an integration site preference near genes, we determined if the number of CrERV loci observed to be within 20 kb of a gene differed from that expected if the distribution was random. There are significantly more observed insertions that fall within 20 kb of the translation start site of a gene than would occur randomly (fig. 4*A*). In contrast, there are fewer intronic CrERV insertions than expected based on our simulations if integration is random (fig. 4*B*). Among, only a sublineage of Lineage A CrERVs (CrERVs at node “a” in fig. 2) is found in closer proximity to genes (bold font in column G of supplementary table S6, Supplementary Material online) than expected if integrations are random (Fisher’s exact test *P *=* *0.002891). Additionally, several recombinant CrERVs (e.g., Lineage A/B recombinant CrERV S10) are close to a gene (supplementary table S6, Supplementary Material online, bold font in column G). Remarkably, four Lineage C CrERVs are within 20 kb of a gene (supplementary table S6, Supplementary Material online, bold font in column G). Our data indicate that integration site preference overall favors proximity to genes but that not all lineages show this preference. However, the history of Lineage C CrERV suggests they could have acquired a different integration site preference through recombination that facilitated recent genome expansion.

## Discussion

The wealth of data on HERVs provides the contemporary status of events that initiated early in hominid evolution. Potential impacts of an ERV near the time of colonization on a host population is thought to be minimal because infection of host germ line by an XRV is a rare event and ERVs that negatively affect host fitness are quickly lost. Potentially deleterious ERVs that are not lost due to reproductive failure can be removed by recombination leaving a solo LTR at the integration site or can suffer degradation presumably because there is no benefit to retain function at these loci; most HERVs are represented by these two states. In addition, humans and other vertebrate hosts have invested extensive genomic resources (Feschotte and Gilbert 2012; Stoye 2012; Zheng et al. 2012) to control the expression of ERVs that are maintained. The dynamics between host and ERV are described as an evolutionary arms race (Daugherty and Malik 2012; Duggal and Emerman 2012). This narrative may underrepresent any contributions of ERVs to fitness as they were establishing in a newly colonized host population. Because there are now several species identified to be at different points along the evolutionary scale initiated by the horizontal acquisition of retroviral DNA it is possible to investigate dynamics of ERV that are not yet fixed in a contemporary species. Considering the numerous mechanisms by which newly integrated retroviral DNA affect host biology, such as by introducing new hotspots for recombination (Campbell et al. 2014), altering host gene regulation (Maksakova et al. 2006; Cohen et al. 2009; Rebollo et al. 2012), and providing retroviral transcripts and proteins for host exaptation (Bénit et al. 1997; Finnerty et al. 2000; Lu et al. 2014; Kawasaki and Nishigaki 2018), colonizing ERVs could make a substantive contribution to species’ evolution. Our research on the evolutionary dynamics of mule deer CrERV demonstrates that genomic CrERV content and diversity increased significantly during a recent retroviral epizootic due to acquisition of new XRV and from endogenization and expansion of recombinants generated between the colonizing and older CrERVs. These data suggest that CrERVs provide a pulse of genetic diversity, which could impact this species’ evolutionary trajectory.

Our analyses of CrERV dynamics in mule deer are based on the sequence of the majority of coding CrERVs in MT273. Of the 252 CrERV loci identified in the MT273 assembly, we were able to reconstruct CrERV sequences from long-insert mate pair and Sanger sequencing to use for phylogenetic analysis at 164 sites; 46 sites were solo LTR and 42 were occupied by CrERV retaining some coding capacity. We complemented phylogenetic analyses with our previous data on the frequency of each CrERV locus identified in MT273 in a population of mule deer in the northern Rocky Mountain ecosystem (Bao et al. 2014; Kamath et al. 2014; Hunter et al. 2017). In addition, we incorporated information on the variable structure of the SU-encoding portion of the retroviral envelope gene, *env*, which typifies retrovirus lineages. The variable regions of retroviral *env* are characterized by insertions and deletion and result from balancing its role in receptor-mediated, cell specific infection while evading host adaptive immune responses (Stamatatos et al. 2009; Murin et al. 2019). The specific pattern of *env* insertions and deletions was particularly useful in characterizing recombination events. By integrating population frequency, coalescent estimation, and the unique structural features of *env* we provide an integrated approach to explore the evolutionary dynamics of an endogenizing ERV.

It is likely that the most recently acquired CrERVs recorded by germline infection reflects an epizootic that occurred coincident with the last glacial period, which ended about 12 ka. The retroviruses that endogenized during this epizootic belong to Lineage A, have open reading frames for all genes and have been recovered by coculture as infectious viruses (Fábryová et al. 2015). The evolutionary history of CrXRV contributing to germline infections over this time period is reflected by several sublineages of Lineage A. Lineage A retroviruses constitute approximately one-third of all retroviral integrations in the genome. Only four of the 50 Lineage A CrERV that we were able to reconstruct did not have a full-length *env*. An important implication of this result is that over the most recent approximately 100,000 years of the evolution of this species, the mule deer genome acquired up to half a megabase of new DNA from germline infections by a new retrovirus, which introduced new regulatory elements with promoter and enhancer capability, new splice sites, and sites for genome rearrangements. Thus, there is a potential to impact host fitness through altered host gene regulation even if host control mechanisms suppress retroviral gene expression. None of the Lineage A CrERV is fixed in mule deer populations (supplementary table S6, Supplementary Material online, column F) so any effect of CrERV on the host will not be experienced equally in all animals. However, none of the Lineage A CrERV is found only in M273 indicating that the burst of new CrERV DNA acquired during the most recent epizootic has not caused reproductive failure among mule deer. These data demonstrate that, in mule deer, a substantial accrual of retroviral DNA in the genome can occur over short time spans of a species history and could impose differential fitness in the newly colonized host population.

The impact of Lineage A CrERV on genomic burden in mule deer is augmented by activation and recombination with other CrERV lineages. Lineage A CrERVs have an open reading frame for *env* but Lineages B–D do not. Lineage B CrERVs all have identical deletions of the extracellular portion of *env* but they also constitute approximately a third of the CrERV in the genome. Although ERV that have deleted *env* are reportedly better able to expand by retrotransposition (Gifford et al. 2012), we show support for an alternative explanation for the prevalence of *env*-deficient Lineage B; complementation with an intact Lineage A CrERV envelope glycoprotein that allowed for germline infection. Complementation is not uncommon between XRV and ERV (Hanafusa 1965; Evans et al. 2009), is well established for murine intracisternal A-type particle (Dewannieux et al. 2004) and has been reported for ERV expansion in canids (Halo et al. 2019). Complementation requires that two different retroviruses are coexpressed in the same cell (Ali et al. 2016). During viral assembly functional proteins supplied by either virus are incorporated into the virus particle and either or both retroviral genomes can be packaged. A recombinant can arise if the two copackaged RNA strands are not identical. Our data show that Linage A and B recombination has occurred several times. A group of CrERV that encode a Lineage B *env* cluster with Lineage A CrERV in a phylogeny based on a partial genome alignment (JN592050: 4,422–7,076 bp). The recombinant breakpoint within this monophyletic group is identical, suggesting that an interlineage recombinant most likely expanded by retrotransposition. Notably, two CrERV in this recombinant cluster were only found in M273, indicating expansion was a recent event. There are other clusters of CrERV with Lineage B *env* affiliated with Lineage A CrERV that have different breakpoints in this partial phylogeny, suggesting that interlineage recombination is not a rare event. Recombination between an XRV and ERV is also a well-documented property of retroviruses (Kozak 2014; Bamunusinghe et al. 2016; Löber et al. 2018). However, the recombinant retroviruses that result are typically identified because they are XRV and often associated with disease or a host switch. Our data indicate that multiple recombination events between Lineage A and B CrERV have been recorded in germline; this in itself is remarkable given that endogenization is a rare event. Thus, both the burden of CrERV integrations and the sequence diversity of CrERV in the mule deer genome increase concomitant with the Lineage A retrovirus epizootic by CrERV interlineage recombination.

**Fig. 5.**
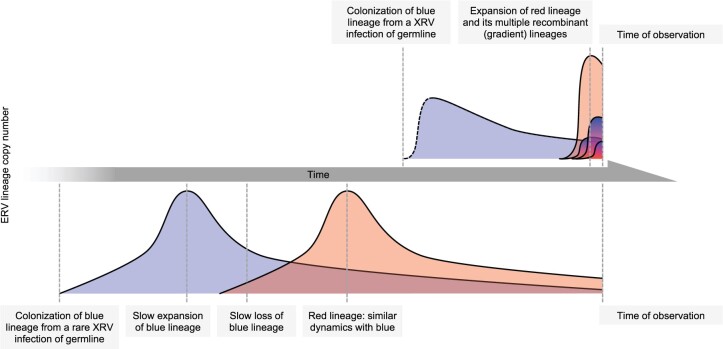
CrERV colonization dynamics. The schematic depicts the dynamics among CrERVs over the period of endogenization of the retrovirus. The prevailing view of ERV endogenization is displayed on the bottom. A rare germline infection occurs in an ancestral species, expands by retrotransposition or reinfection, and declines due to loss from the population, conversion to solo LTR, or accumulation of deletions. Few intact ERVs survive to contemporary species. In mule deer, we have a close-up view of the dynamics surrounding colonization by this gammaretrovirus in a contemporary species. For simplicity, interactions between only two lineages are displayed, with Lineage A represented by red. Our data demonstrate continual colonization by CrXRVs over the last 150,000 years causing a rapid increase in CrERV burden in mule deer genomes. Activation of and recombination with existing CrERV increase both the number of CrERV loci and CrERV genomic diversity. The initial infection dynamics for the first (blue) colonizing CrERV are indicated in dotted lines because there are no data to support a rapid rise, as seen with CrERV Lineage A, but it is possible that this rapid expansion could have occurred at each new colonization event.

Recombination is a dominant feature of CrERV dynamics and quite evident in the evolutionary history of Lineage C CrERV. Our phylogenetic analysis places the ancestor of Lineage C CrERV at 500 ka and indeed, Lineage C and Lineage D, which is estimated to be the first CrERV to colonize mule deer after splitting from white-tailed deer (Elleder et al. 2012; Kamath et al. 2014), share many features in *env* (supplementary fig. S2, Supplementary Material online). Consistent with a long-term residency in the genome, many Lineage C CrERVs are found in most or all mule deer surveyed. Although these data would fit the paradigm that a single XRV colonized the genome and recently expanded by retrotransposition, our analysis shows that all Lineage C CrERV are recombinants of a Lineage A CrERV and a CrERV not recorded in, obscured, or lost from contemporary mule deer genomes. Hence the resulting monophyletic lineage did not arise from retrotransposition of an ancient colonizing XRV. Rather, the contemporary makeup of the mule deer genome is dominated by Lineage A dynamics with other lineages (fig. 5). We propose that the following sequence of events occurred: 1) activation of CrERVs in any cell by a lineage A CrERV/CrXRV infection/expression, 2) recombination of copackaged CrERV genomes during reverse transcription in a newly infected cell, 3) infection of germline by interlineage recombinant, and 4) expansion of the recombinant. CrERV with multiple recombination sites from different lineages are evidence that these new recombinant loci continue to be expressed, recombine, and enter germline. It is noteworthy that all CrERV clusters that recently expanded are interlineage recombinants sharing a common breakpoint, suggesting that either the host genome location or new retrovirus genome properties facilitated expression. We also note that some of the deletions we document in Lineages B–D are not from slow degradation in the genome but rather are a consequence of errors during reverse transcription, as was recently reported for Koala retrovirus (Löber et al. 2018).

Our study provides a unique window on the burst of genomic diversification a host population can experience as a new ERV lineage endogenizes. The CrERV genomic burden in mule deer increases notably during colonization by new acquisitions and pulses of retrotransposition or reinfection of interlineage recombinants. CrERV sequence diversity also increases consequent to multiple interlineage recombination events. This pulse of newly acquired CrERV loci in the genome increases host genetic diversity and hence has the potential to affect host fitness. Indeed, several of the recombinant Lineage C CrERVs showing recent expansion are within 20 kb of a gene, including one that is fixed in our sampled population. This raises the question as to whether there is a fitness effect at these loci that is in balance with expression of the retrovirus. Because both interlineage recombination and endogenization are rare events, it is remarkable that so many of the events marking the dynamics of retrovirus endogenization are preserved in contemporary mule deer genomes. Although our study only investigates germline infection, it is likely that the dynamics we describe here also resulted in infection of somatic cells. It is worthwhile to consider the potential for ERVs in other species, in particular in humans where several HERVs are expressed, to generate novel antigens through recombination or disruptive somatic integrations that could contribute to disease states.

## Materials and Methods

### Sequencing

Whole-genome sequencing was performed for a male mule deer, MT273, at ∼30× depth using the library of ∼260 bp insert size, ∼10× using the library of ∼1,400–5,000 bp insert size, and ∼30× using the library of ∼6,600 bp insert size. 3′ CrERV-host junction fragment sequencing was performed as described by Bao et al. (2014). 5′ CrERV-host junction fragment sequencing was performed on the Roche 454 platform, with a target size of ∼500 bp containing up to 380 bp of CrERV LTR.

### Assembly and Mapping

The draft assembly of MT273 was generated using SOAPdenovo2 (Luo et al. 2012) (supplementary file S1, section 2a, Supplementary Material online). WGS data were then mapped back to the assembly using the default setting of bwa mem (Li and Durbin 2009) for further use in RACA and CrERV reconstruction. RNA-seq data were mapped to the WGS scaffolds using the default setting of tophat (Trapnell et al. 2009; Kim, Pertea, et al. 2013). 3′ junction fragments were clustered as described in Bao et al. (2014). 3′ junction fragment clusters and 5′ junction fragment reads were mapped to the WGS assembly using the default setting of blat (Kent 2002). A perl script was used to filter for the clusters or reads whose host side of the fragment maps to the host at its full length and high identity. 5′ junction fragments were then clustered using the default setting of bedtools merge.

### Reference-Assisted Chromosome Assembly

Synteny based scaffolding using RACA was performed based on the genome alignment between the mule deer WGS assembly, a reference genome (cow, bosTau7, or Btau7), and an outgroup genome (hg19). Genome alignments were performed with lastz (Harris 2007) under the setting of “–notransition –step = 20,” and then processed using the UCSC axtChain and chainNet tools. The mule deer–cow–human phylogeny was derived from Bininda-Emonds et al. (2007) using the “ape” package of R.

### CrERV Sequence Reconstruction

CrERV locations and sequences were retrieved based on junction fragment and long-insert mate pair WGS data. The long-insert mate pair WGS reads were mapped to the reference CrERV (GenBank accession number JN592050) using bwa mem. Mates of reads that mapped to the reference CrERV were extracted and then mapped to the WGS assembly using bwa mem. Mates mapped to the WGS assembly were then clustered using the “cluster” function of bedtools. Anchoring mate pair clusters on both sides of the insertion site were complemented by junction fragments to localize CrERVs. Based on the RACA data, CrERVs that sit between scaffolds were also retrieved in this manner. CrERV reads were then assigned to their corresponding cluster and were assembled using SeqMan (DNASTAR). Sanger sequencing was performed to complement key regions used in CrERV evolutionary analyses. All reconstructed CrERV sequences used in the phylogenetic analyses are included in supplementary file S2, Supplementary Material online, in fasta format.

### CrERV Evolution Analyses

CrERV sequences of interest were initially aligned using the default setting of muscle (Edgar 2004), manually trimmed for the region of interest, and then realigned using the default setting of Prank (Löytynoja and Goldman 2005). Lineage-specific regions are manually curated to form lineage-specific blocks. Models for phylogeny were selected by AICc (Akaike Information Criterion with correction) using jModelTest (Posada 2008). Coalescent analysis and associated phylogeny (fig. 2) was generated using BEAST2 (Bouckaert et al. 2014). In the coalescent analysis, we used GTR substitution matrix, four Gamma categories, estimated among-site variation, Calibrated Yule tree prior with ucldMean ucldStddev from exponential distribution, relaxed lognormal molecular clock, shared common ancestor of all CrERVs 0.47–1 Ma as a prior (Elleder et al. 2012; Kamath et al. 2014). Maximum likelihood phylogeny in figure 3 was generated using PhyML (Guindon and Gascuel 2003) using the models selected by AICc and the setting of “-o tlr -s BEST” according to the selected model.

### CrERV Spatial Distribution

We simulated 274 insertions per genome to approximate the average number of CrERVs in a mule deer (Bao et al. 2014). The simulation was performed 10,000 times on three genomes: the mule deer WGS scaffolds, cow (Btau7), and human (hg19). Distance between simulated insertions and the closest start of the coding sequence of a gene was calculated using the “closest” function of bedtools, and the simulated insertions that overlap with a gene were marked with the “intersect” function of bedtools. Number of simulated simulations that are within 20 kb or intronic to a gene was counted for each of the 10,000 replicates. Counts were then normalized by the total number of insertions and plotted using the “hist” function of R.

## Supplementary Material

Supplementary data are available at *Molecular Biology and Evolution* online.

## Supplementary Material

msab252_Supplementary_Data
