# Supplementary material for: Recombination Marks the Evolutionary Dynamics of a Recently Endogenized Retrovirus
Source: Mol Biol Evol. 2021 Sep 4;38(12):5423–36. doi: 10.1093/molbev/msab252 (PMC8662619; doi:10.1093/molbev/msab252)
Supplement: msab252_Supplementary_Data [file msab252_supplementary_data.zip › Figure S1 Poss MBE-21-0328.pdf]

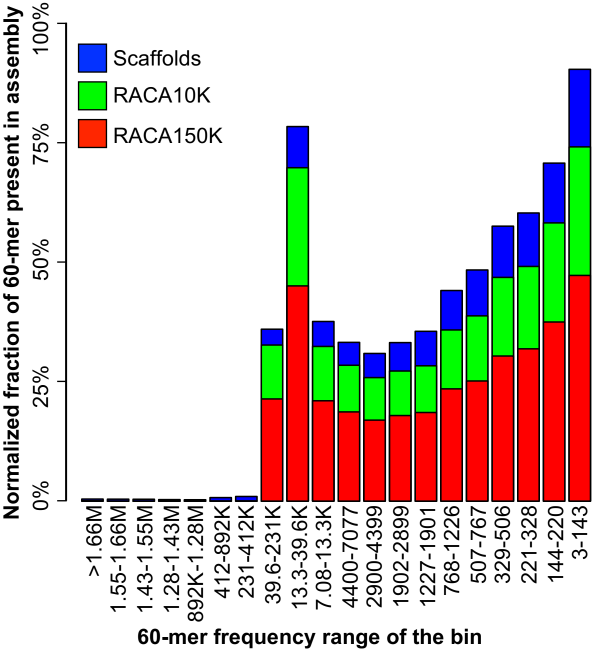

Figure S1. *K-mer* representation of missing data in assemblies. *60-mers* were generated from the raw paired-end WGS, then ranked and classified into 20 bins containing equal number of *60-mers*. Sorted by the *60-mer* frequency range, bars represent genome repeats from high copy number (left) to low copy number (right). Color bars show percent of all *60-mers* present in the scaffolds/contigs (blue), RACA10K (green), and RACA150K (red) assemblies. *K-mer* counts were based on the total number of *k-mers* (*k-mer* of frequency *n* were counted *n* times). *K-mers* in the raw sequencing data were normalized based on sequencing depth, genome ploidy, read length and *k-mer* length, so that the *k-mer* fractions reflect the proportion of *k-mers* that are present in each assembly compared to the raw sequencing data. All bars start from 0% instead of being stacked. Numbers beneath each bar indicates the range of frequency of *60-mers* in the raw paired-end genome sequencing data in that bin. M: million, K: thousand.
