# Supplementary material for: Recombination Marks the Evolutionary Dynamics of a Recently Endogenized Retrovirus
Source: Mol Biol Evol. 2021 Sep 4;38(12):5423–36. doi: 10.1093/molbev/msab252 (PMC8662619; doi:10.1093/molbev/msab252)
Supplement: msab252_Supplementary_Data [file msab252_supplementary_data.zip › Figure S2 Poss MBE-21-0328.pdf]

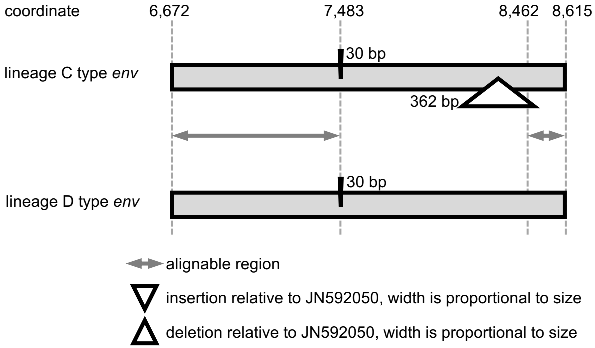


**Figure S2. Depiction of Lineage C and D alignment.** Coordinates shown are relative to JN592050. Triangles representing insertions and deletions are anchored at the center of the event.
