# Supplementary material for: Recombination Marks the Evolutionary Dynamics of a Recently Endogenized Retrovirus
Source: Mol Biol Evol. 2021 Sep 4;38(12):5423–36. doi: 10.1093/molbev/msab252 (PMC8662619; doi:10.1093/molbev/msab252)
Supplement: msab252_Supplementary_Data [file msab252_supplementary_data.zip › Figure S3 Poss MBE-21-0328.pdf]

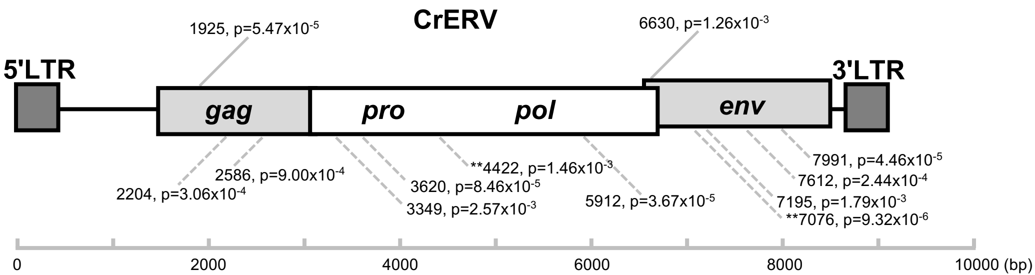

Figure S3. Diagram of CrERV recombination breakpoints. Gray lines point at the key recombination breakpoints on the CrERV. Text box connected to the gray lines indicate the coordinate and adjusted p-value of the breakpoint. Solid gray lines indicate breakpoints of recombinant lineages; dashed gray lines indicate additional breakpoints detected by testing the alignment of reference non-recombinant and candidate recombinant CrERVs. All coordinates are relative to GenBank entry JN592050. Double star (**) indicates breakpoints used in the Lineage B recombinant analysis.
