# Supplementary material for: Recombination Marks the Evolutionary Dynamics of a Recently Endogenized Retrovirus
Source: Mol Biol Evol. 2021 Sep 4;38(12):5423–36. doi: 10.1093/molbev/msab252 (PMC8662619; doi:10.1093/molbev/msab252)
Supplement: msab252_Supplementary_Data [file msab252_supplementary_data.zip › Figure S4 Poss MBE-21-0328.pdf]

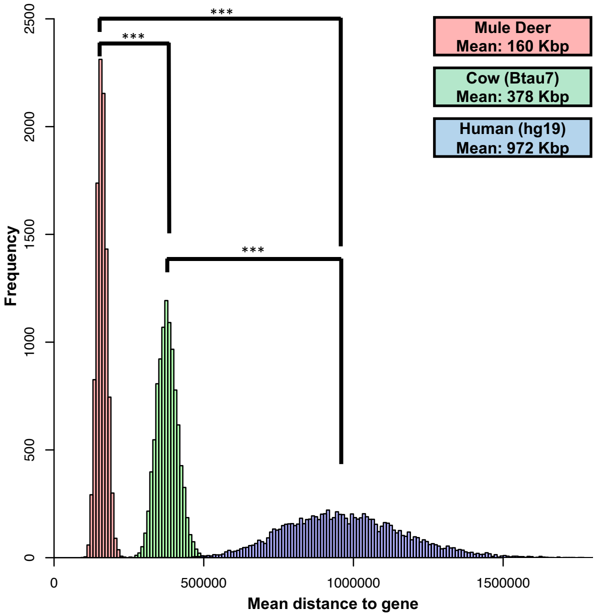


**Figure S4. Distribution of simulated mean distance to gene per replicate in mule deer, cow and human genome.** Distribution of mule deer, cow and human are colored in red, green and blue respectively. Mann-Whitney U test p-values in all three comparisons are less than $2.2 \times 10^{-16}$.
