# Supplementary material for: Recombination Marks the Evolutionary Dynamics of a Recently Endogenized Retrovirus
Source: Mol Biol Evol. 2021 Sep 4;38(12):5423–36. doi: 10.1093/molbev/msab252 (PMC8662619; doi:10.1093/molbev/msab252)
Supplement: msab252_Supplementary_Data [file msab252_supplementary_data.zip › Response to reviews Poss MBE-21-0328.docx]

1.1. The text refers to lineages B1 and B2, but Fig 2 only lists the B and B1 nomenclature.

>> We have changed the nomenclature in Fig 2 to B*i* and B*ii*. Both are now labeled. The *env* structure of B*ii* can’t be determined accurately because the 5 CrERV that fall in this sublineage all had partially missing data in env. This is now indicated in the text page 9 and Figure 2 legend.

1.2. Figure 2 generally is a bit confusing. The lineage nomenclature is assigned to the env genes and it is not immediately obvious it applies to the viruses in their entirety. This can be clarified with improved labeling.

>>We have made several changes to Figure 2 in response to reviewers and editors comments. Briefly, the phylogeny is based on the alignment of the coding region spanning 1,477-8,633 bp [referenced to the position of a full length CrERV, JN592050]. The variable regions of *env* were manually blocked and included in the analysis. This is possible because the *env* gene of all members of a lineage have the same structure of conserved and variable regions, which is depicted in the diagram. We have also clarified frequency information as requested. The manuscript text page 8 and Figure 2 legend contain this information.

1.3. In constructing the phylogenies the authors describe using sequence regions that lack signatures of recombination. The methods for identifying recombination-free regions are only well-described in the supplemental methods. It would be helpful to provide some information on the approach to this in the body of the text.

>>We have included a statement of the approach to detect recombination in the text and referenced the supplementary material [page 8] as the reviewer requests.

1.4 LinD env genes. The authors describe a shared insertion - depicted in Fig 2, and sequence homology. However the sequence homology is not shown nor does the text refer to a supplemental figure containing this information. These data are necessary to determine whether the degree of sequence homology supports an origin of LinC involving a LinD-like virus.

>>We have added a graphic showing the alignment of LinC & D env [Figure S2].

1.5. LinC ERVs seem closer to fixaton than LinA ERVs, based on Fig 2. Why is this true if LinC are a recombinant of A/D ERVs, suggesting that LinC actually appeared after the LinA epizootics and endoginezation events?

>> This is an important point and we have clarified in the text [page 12]. Briefly, of the four putative non-recombinants, 1 is found in all sampled mule deer and the other three in 43-68% of animals. Members of the oldest lineage A subgroups are also found in 40-75% of animals. We hypothesize that the lineage C CrERV found in all animals could have been swept to high prevalence rapidly because of a selective advantage conferred by its close proximity to a gene. Thus, recombination of a member of the first wave of Lineage A CrERV with an existing older CrERV [related to lineage D or absent from the genome] is plausible. Most CrERV with this particular structure of *env* [insertion c and/or deletion e] show multiple sites of recombination; the recombinant partner is most frequently Lineage A. The data supporting an earlier colonization of lineage C ancestors are the older estimate of divergence of the putative ancestor and shared features of *env* variable and conserved regions with that of Lineage D [new Figure S2]. Lineage C ancestry is clearly complex and represents the problems with age estimates in retroviruses with high levels of recombination, which characterize this lineage.

1.6. The manuscript would be greatly enhanced by a diagram/schematic showing the complicated evolutionary histories of these CrERVs. For example, the recombination findings re LinC would seem to upend the phylogeny presented in Fig 2. However, this is never displayed in any way making it difficult to visualize what this would look like.

>> We have included Figure 5 and expanded the discussion to summarize these events. A main finding of our study is how dynamic [and misleading] ERV evolution can be during the colonization of a new retrovirus lineage when there is extensive recombination among ERVs; we are fortunate to have a glimpse into these short-term and complex dynamics in mule deer.

1.7. With respect to LinC distribution, the authors speculate recombination may have altered the insertion site preference. Given the apparent recent expansion of LinC via retrotransposition, is it possible that insertion site preference is

>>This was truncated in the comments but if the reviewer was wondering if the recombination could have affected insertion site preference, we agree that this is a possibility. This is included in the results page 13.


Reviewer: 2

Comments to the Author
This paper describes the genomic sequencing and assembly of the genome of a mule deer and characterizes the phylogeny and distribution of cervid endogenous retroviruses that endogenized in this animal after it was split from its sister species in the same genus.  This describes a more complete description of the individual CrERVs in a single genome than previously reported, and the individual CrERVs were placed into 4 phylogenetic lineages.  Evidence was presented of recombination among the groups, which was described in a more limited way previously.  Of particular interest is the successful assembly of individual ERVs, which is something that has presented problems for assemblies of other genomes.  A few comments:

2.1.    It would be helpful if the authors provided some more basic information on the nature of these ERVs. Previously identified CrERVs were identified as gammaretroviruses, but the point is made on line 272 that some lineages have different env genes – are all gammas?  A point is made on lines 251-2 about the consistent env variable domains, but what about the rest of env, and in particular the TM.  While the authors excluded env in phylogenetic analysis due to its variability, the TM is more conserved and useful for phylogenetics.

2.1a. *A previous paper, Bao et al.,2014, also described CrERV recombinants but it is not entirely clear how the structures identified in the current analysis compare with what was previously identified.*

>>We have clarified these points in the text (page 8 Evolutionary history of CrERV). Briefly,

Yes, all CrERV are gammaretroviruses.

The surface unit of all retrovirus’ envelope proteins {SU} contains variable and conserved regions. The regions termed variable have multiple insertions or deletions and are flanked by conserved regions, which have sequence variation but are alignable. [Crystal structures of HIV *env* demonstrated that the variable regions are often loops or disordered regions where length variation does not affect the core structure.] The conserved regions of SU and all of TM have sequence differences but can be aligned. Viral subtypes sharing a recent ancestry typically have a similar structure of *env* variable regions. Thus we manually blocked the variable regions [in this way we don’t infer that those regions in different lineages evolved from a common ancestor] and included them with the alignable conserved regions for phylogenetic analysis.

>>Bao et al 2014 reports on statistical models for more accurately utilizing junction fragment data. Kamath et al, did utilize the sequence of 14 partial CrERV sequences and reported a signature of recombination in these. It is difficult to directly compare the results because each CrERV was obtained from a different animal, only the 3’ portion of the genome was used, and larger regions of the partial genome sequence were omitted from the alignment in Kamath et al. However, the divergence dates for our Lineage D [Kamath et al, Lineage I] both approximate 700 MYA [in text and referenced page 10]. The divergence of the youngest of Lineage A [Kamath et al Lineages III and IV] is estimated by both methods at 50KYA. We now mention this concurrence and add the reference in the text [page 9]

2.2.    Authors performed molecular clock analysis of the CrERVs  in this genome as presented in Figure 2. In a previous publication from this group (Kamath et al. 2013 J. Hered) this same type of analysis was used and compared to estimates based on LTR dating, but they did not do that here.  As various conclusions in the current study about the CrERVs are based on their ages, it would be worth comparing estimates from the two methods where possible.

>>Kamath et al. 2013 J. Hered reported that only the oldest [Lineage D] CrERV have any differences between 5’ and 3’ LTRs and therefore LTRs were not useful for dating the majority of CrERV. Our data on LTR sequences are consistent with that finding. Only 6 of the 164 sequences used had confirmed sequence differences between the 5’ and 3’ LTR. In addition, because of the extensive recombination that we find in these sequences we based our analyses on complete coding regions.

We have added this information to the results Establishing the location and sequence at CrERV loci, top of page 8]

2.3.    I’m not convinced about the value gained from simulating random insertions of CrERVs in the mule deer genome. None of the retroviruses studied so far have a completely random integration profile, and several genome-scale studies have been done for various retroviruses to determine which sites are preferred by each of these. I believe authors should cite some of these studies and mention their results.

>> We agree that there is no expectation for gammaretroviruses to have a random distribution in the genome. We have clarified this in the text and studies are now cited [page 13]. Briefly, we used a random distribution to determine how our draft assembly, which omits repetitive sequence that affects spacing of genes, compared to a complete genome (human) and a draft genome that is largely complete (cow) in determining proximity to genes. Our data demonstrate that results are biased towards closer gene proximity on our draft genome, as expected. Thus, we used the 10,000 random distributions the frequency with which a random point occurred within 20kb of a gene on our draft mule deer genome and compared this to observed number of CrERV in this interval. We were able to reject the hypothesis that integrations are random for some groups of CrERV, but not all, suggesting that the different phylogenetic lineages or a CrERV derived by recombination could have new properties affecting integration site preference.

2.4.    NCBI database contains two mule deer genome assemblies (GCA_004115125.1 and GCA_003697985.1) from two different subspecies. I think it would be important for the authors to compare the location and sequence of the CrERVs found in the individual they sequenced to the genome assemblies in the database. This would not only be significant to check for confirmation of their results but also may give insights into whether some of these CrERVs are only found in the individual or subspecies they sequenced.

>>We agree that comparative analyses would be valuable and could reveal if the high degree of insertional polymorphism contributes to genome structure variation or phenotypic properties now that mule deer genomes are becoming available. However, as we note in the text [Establishing the location and sequence at CrERV loci page 7], only 3 CrERV loci were assembled in our genome draft. We can only confirm the location and reconstruct the CrERV sequences using long insert mate pair data and from our previous detailed junction fragment analyses of 63 mule deer. In addition, we used RACA to provide a chromosome level representation of the mule deer genome to reconstruct CrERV that break scaffolds. Because the new genomes are generated with short read sequence identifying presence or absence of a CrERV would be difficult and determining the sequence at a locus not feasible.

We have previously determined [Bao et al] the frequency of all insertions in a population of mule deer in the northern Rockies Ecosystem and report in this paper those insertion sites that only occur in MT273 [Table S6]. From these previous analyses we reported that 80% of CrERV integrations are found in 4 or fewer animals; providing support for the relatively recent acquisition of these gammaretroviruses in the mule deer genome.

2.5.    Line 62 should reference the original sequencing of the human and mouse genomes which provided these estimates.

>>Done

2.6.    Line 94-99.  Retroviral DNA is called a provirus after integration, and is referred to as an ERV if the provirus is generated in a germline cell and transmitted to subsequent generations.

>>We have made this correction [pg 3]


Reviewer: 3

Comments to the Author
Yang et al

This manuscript by Yang and colleagues describes the most thorough examination of endogenous retroviruses (ERV) of mule deer to date. While all vertebrate species’ genomes harbor ERV, mule deer are among a small subset of species that are of particular interest because of evidence suggesting that some lineages of ERV have recently entered the germline (and could, at least in theory, still be capable of new insertions): thus, CfERV in the mule deer germline join a handful of other organisms in being particularly useful for understanding the early stages of ERV acquisition and fixation in a natural population. The published data for recent endogenization in mule deer is already strong; this study extends and deepens these studies, in part by generating a mule deer genome assembly that can be combined with population data. I don’t have any major concerns with the analyses, but would like the authors to address some minor issues, in particular in the discussion.

3. 1A summary table would be very useful in the main article - something that includes the lineages, the # of loci in each, the env status, age range, that sort of thing. It is easy to get confused when reading the main text, and readers shouldn’t have to consult supplementary figures for the basic info being presented in the main text.

>>Table 1 with the suggested information is now in the manuscript.

3.2 Figure 2 is confusing and difficult to interpret. First, since the taxon labels are written out alphanumerically, why not include the actual frequencies next to each branch as well? This would be a lot easier to understand than the greyscale legend (most of which are very similar shades of black and dark grey). Second, could the authors please consider using a different labeling scheme for the deletions, since capital letters are already being used for the lineages? Having a lineage “A” and “B” and “C” and also deletions labeled as “A”, “B” and “C” is very confusing. Perhaps deletions could be numbered, or labeled in lower case letters or roman numerals? Third, if lineage B contains B1 and B2, what is the rest of lineage B? Might it be better to number the main group as well (e.g. B1, B2, B3, something like that)?

>>Figure 2 has been changed to reflect the reviewer suggestions. Specifically, frequencies are indicated, insertions and deletions are in small letters, and the distantly related lineages to Lineage B are indicated as B*i* and B*ii*.

3. 3 is this an unrooted tree that appears rooted, or was it rooted (legend should indicate). If rooted, how did the deletion “C” get restored in lineages A & B??

>> This is an unrooted tree, now indicated in the Figure 2 legend.

3.4 Figure 3 - do the collapsed branches in orange include the sublineages of B (e.g., B1 and B2)? If so, why not indicate these?

>>B*ii* is included in Figure 3 and is now labeled. It sits on a long branch indicative of its outlier origin. There are no B*i* or B*ii* CrERV within the collapsed clusters.

3.5. Lines 94-96 - “provirus” refers to the integrated form of the viral DNA, whereas as worded this sentence implies that reverse transcribed DNA is “provirus”. Should re-word this for accuracy.

>>Done.

3. 6. line 98 - technically, might want to say “all nucleated cells”. Enucleated cells (e.g. RBCs in many mammals) won’t contain ERV.

>>Done

3. 7. Line 175 - it is recommended to use “identity” or “similarity” rather than “homology”, to avoid confusion. The term homology/homologous becomes important when referring to ERV loci that arise from the same integration event and are in the same site in related taxa or species.

>>We have clarified as suggested

3.8. Line 184-5 - claim that repetitive elements break scaffolds could use a reference.

>>Done

3.9. Lines 278-9 - same as figure 2. The text says there are two groups of CfERV affiliated with lineage B, but B1 and B2 don’t appear to account for all of lineage B. The text implies two groups, the tree indicates at least three.

>> We have clarified on page 9 that the main lineage B CrERVs all share the same *env* structure and arise from node g. B*i* appears to be the ancestor to lineage B based on its prevalence and complete *env* structure. Lineage B*ii* is a distant relative to Lineages B and Bi. We speculate that this CrERV evolved in a different species and recently infected mule deer, accounting for its unusual *env* and phylogenetic position.

3.10. Lines 309 and 482 refer to retroviral epizootics, but if some of these insertions arise by retrotransposition or complementation, would these be “epizootics”? Also, 309 refers to unique env structure, but aren’t some of these env defective (in which case, could they really represent an independent epizootic?). Maybe some clarifying text would help here.

>>The reviewer is correct that individual integration sites within a lineage can arise by retrotransposition, reinfection, or complementation (for defective genomes). However, the new genetic material that gives rise to the phylogenetically distinct lineages are typically from exogenous infection. We agree with the reviewer that given the extensive amount of recombination we document here, particularly with Lineage C, it is possible that the immediate ancestor to Lineage C never existed and that these CrERV are totally derived from multiple rounds of recombination with ancient [existing in other species] ERV (we now mention this possibility in Results and Discussion). However, for Lineage D, the absence of integrations shared with white-tailed deer indicates that this lineage was acquired by infection post-speciation. Similarly, Lineage A is clearly capable of infection and reflects the phylogenetic pattern expected for an evolving infectious retrovirus that has been persistent in mule deer populations in the recent past and whose members were periodically preserved in the mule deer genome. Lineage B appears to have originated from Lineage B*i* [likely from an error in reverse transcription, which deleted a portion of *env*) and expansion of the *env*-less Lineage B was facilitated by complementation with Lineage A as well as retrotransposition. We have clarified these points in Results page 12 and the Discussion.

3.11. Line 454 - complimented or complemented?

>>Always hopeful for a compliment. Thanks for the careful read. Corrected.

3. 12. Line 500 - “During viral assembly functional genes supplied by either virus are incorporated…” should be “During viral assembly functional proteins supplied by either virus are incorporated…”.

>>This has been corrected.

3.13. Discussion in general - the term retrotransposition is used frequently (e.g. lines 492, 509, 510, 530, 536, 544) but it’s not clear that this is what the authors mean. As they discuss earlier in the text, retrotransposition refers to re-insertion of proviral sequences in the same cell, whereas the term is being used frequently where it is also possible or even likely that re-infection is what occurred (i.e., by recombination or complementation to produce an infectious, extracellular particle).  In fact, the authors argue that Env complementation occurred in some cases - thus, retrotransposition would not be the correct term.

>>This is a good point and we have clarified in the text. Briefly, our rationale behind using retrotransposition of LinC is that this CrERV is defective in most genes and the members of the expanded group are essentially identical. Thus, the CrERVs forming a closely related group are likely the result of expansion by retrotransposition. We expect that if complementation allows for infection by a defective genome, that the likelihood of recombination would increase. We have evidence of such multiple recombinants, but they are usually outliers in the phylogeny because the breakpoints are all different. We point this out in Fig 3 and have included additional text to clarify. But the reviewer is absolutely correct, we can only tell complementation IF we see a recombinant and the absence of recombination does not rule this out. Thus, either process could result in the recent expansion of related CrERV. Thanks for raising the point.
